# Our Institutions

**Published:** 1895-10

**Authors:** 


"Our institutions of learning should take heed that the manual skill of their graduates does not suffer on account of increased theoretical training. There is no valid reason why the two conditions, manual and mental training, should not be cultivated hand in hand. To maintain our national preeminence as conservators of the natural teeth we must, without lowering our vaiue of scientific knowledge, retain the highest regard for operative skill."—"From Report of the Committee on Practice, Dental Society of the State of New York."

---

### Monthly Summary.

